# Supplementary material for: Are carbon emissions trading and green financial instruments synergistic? -Comprehensive quantitative research based on content analysis
Source: PLoS One. 2024 Mar 7;19(3):e0298601. doi: 10.1371/journal.pone.0298601 (PMC10919601; doi:10.1371/journal.pone.0298601)
Supplement: S1 Data — (ZIP) [file pone.0298601.s001.zip › Data/地级市Z_F工作报告.zip!/║╙▒▒/▒ú╢¿╩╨/└┤╫╘╣1⁄2╓┌║┼í╛┬φ┐╦╩2╛▌═°í┐_2007.docx]

各位代表：

我代表保定市人民政府向大会作工作报告，请予审议，并请市政协各位委员和列席会议的同志提出意见。

一、过去一年工作回顾

2006年是“十一五”开局年。在省委、省政府和中共保定市委的正确领导下，我们高举邓小平理论和“三个代表”重要思想伟大旗帜，全面落实科学发展观，高扬“树正气、讲团结、求发展”主旋律，深入开展“学树保促”活动，团结和依靠全市人民，立足“打基础、增后劲、求发展”，奋发图强，扎实工作，较好地完成了市十二届人大四次会议确定的各项任务，实现了起好步、开好局的目标。总结去年工作，可以简要用四句话来概括：

第一，科学谋划推动科学发展。科学谋划是成功的前提，只有在谋划上多下功夫，才能避免“建设性浪费和破坏”，才能推动科学发展。为全面落实“十一五”规划，我们按照科学发展观的要求，结合我市实际，加强了对具体工作思路和措施的深入谋划，提出了保定发展要靠“保定制造”、“保定文化”双轮驱动，要靠众人划桨开大船、实施“强县兴市”战略；统筹区域发展要“做大中心、发展南北、保护两翼”；明确了产业发展要着力构建“南车北电东纺西绿城文”[1]的空间布局；打造文化保定要抓好“一纲一力四目”[2]；县域发展要突出“四项重点”[3]；强调新农村建设要“通进去、走出来、化起来”[4]，城市建设要往细里做、往精致里做、往群众身边做以及城乡规划管理全覆盖[5]、职业教育全方位[6]等一系列工作思路，出台了相应的规划、政策和措施，取得了初步成效，全市经济社会发展呈现积极变化。

一是主导产业快速成长，发展支撑力增强。谋划实施了“中国电谷”建设项目95项、总投资404亿元，新能源和能源设备制造业强势发展，销售收入、利税分别增长50%和65.6%。汽车及零部件业实现新跨越，长城推出3款轿车，结束了河北无轿车生产的历史。全年全市整车销售28万辆，全行业销售收入200亿元，分别增长30.2%和27.7%。全市汽车出口突破5万辆，居全国第一。纺织服装业整合升级步伐加快，销售收入和利税分别增长28%和36%。这三大战略支撑产业占入统工业增加值比重由30.9%上升到33.7%。全市投资亿元以上项目119个，5亿元以上41个，比上年分别增加19个和16个。入统工业增加值268.7亿元，利税97.3亿元，分别增长17.6%和20.6%；工业经济效益综合指数同比提高9.9个百分点。

二是县域经济基础进一步夯实，发展活力增强。县城经济聚合、辐射带动作用得到重视。新规划建设工业园区12个，规模以上民营企业新增100家。农业产业化取得突破，新上了定州伊利、望都蒙牛、顺平汇源、高碑店康师傅等一批产业化龙头项目。粮食生产稳定增长，蔬菜等优势农产品标准化生产面积扩大，果品产量突破100万吨，牛奶加工能力增长100%。

三是改革开放步伐开始加快，发展动力增强。国企改革开始攻坚，13户中小企业进入破产程序，市属大型企业集团改革逐步深入，国资监管工作得到加强。行政管理体制改革稳步推进，市行政服务中心正式运行。实施城乡规划管理体制大调整，在全国率先推行城乡规划管理全覆盖，实行了城市规划审议决策票决制。理顺了文物管理体制。粮食流通等各项改革都取得新进展。金融环境有所改善，设立了市金融证券工作办公室，城市信用社组建城市商业银行完成增资扩股，农村信用社改革不断深化，与省农发行签订60亿元的合作协议，加强了与省开行合作。主动走出去，在长三角、珠三角、东北和韩国、欧洲等地成功举办了专题招商推介活动，新签约外资项目44项。我市和长安集团签署了长期战略合作协议；中兴与美国ZXNA公司合作建立汽车出口生产基地；天威英利与西班牙安讯能公司合作建设世界最大的太阳能发电站。全市实际利用外资1.78亿美元，增长37.4%；出口创汇16.3亿美元，增长48.2%。

四是各项社会事业全面进步，发展协调性增强。文化大市建设起步上路，市政府与河北大学签订了战略合作协议。在北京等地开展了大规模的“迎奥运游保定”推介活动，“跟着老电影去旅行”入选全国十佳旅游线路，白洋淀5A级景区创建工作接受国家验收，全市旅游接待人数、综合效益居全省第一。“山花工程”助学义演、“古城大风歌”等文化活动丰富多彩。市档案馆晋升为省一级馆。科技创新走在全省前列，高新区被命名为“国家科技兴贸出口创新基地”。职业教育得到重视和加强，各类教育协调发展，保定师专顺利升本，改造中小学危房10.4万平方米，高考本科二批以上上线人数保持全省第一。医疗救治和疾病防控体系进一步健全，公共卫生服务能力增强。全民健身活动蓬勃开展，竞技体育再创佳绩，保定籍运动员亚运会夺金数量占全省一半，新增加1名世界冠军。圆满完成了人口与计划生育年度目标，低生育水平保持稳定。节能降耗和污染物减排工作全面加强。安全生产形势基本平稳。国防教育、民兵预备役和民政、统计、监察、审计、民族宗教、外事侨务等各项工作都取得了新成绩。

第二，破解难题凝聚人心力量。针对长期困扰我市发展稳定的突出问题，特别是定州“6?11”事件影响，我们从破解难题入手，努力为发展扫清障碍、营造环境、凝聚力量。采取安排就业、享受低保等办法，取缔了市区近万辆经营性三轮车，并借机实现出租车扩容，用扩容收益收购三轮车，解决了这一影响交通秩序和市容环境的老大难问题；依法拆违、有情操作，开展了拆违第一战役，拆除市区14条主要街道两侧违法建设150多万平方米，做到了“零强拆、零上访、零事故”，人民日报头版作了专题报道；妥善解决了拖延近一年的保沧高速南沟头段进场问题，实现了全线无障碍施工；仅用两个多月时间就完成了南水北调保定段8.8万亩永久和临时占地的征迁、补偿工作；开展了白洋淀污染综合整治战役，关停了400多家污染企业；启动了长期影响市区大气质量的储灰场搬迁；备受关注的天威英利三期工程顺利进场施工；妥善解决了拖延多年的大慈阁修缮和开放问题；完成了省挂号数年的4处重大火灾隐患整改；及时果断处置了国内罕见的大沙河重度污染突发事件；集中治理了市民反映强烈的狗患问题，等等。实践使我们深深体会到，保定人民是顾全大局、甘于奉献的人民；广大党员和干部是勇于承担责任、敢于攻坚克难，关键时刻拉得出、用得上、能打硬仗的队伍。难题往往不是难在群众和干部身上，而是难在我们领导心上，只要敢于不给自己留后路，敢于碰硬，全市上下一条心，就没有解不开的难题，就没有闯不过的难关。

第三，关注民生促进社会和谐。深入实施“民心工程”，积极推进和谐保定建设。实施了零就业家庭就业再就业援助工程[7]，全市城镇新增就业5.6万人，下岗职工再就业2.7万人，城镇登记失业率比省定目标低0.57个百分点。启动了农民健康保健工程，125万农民参加新型农村合作医疗，在全国率先设立了81个农民健康“流动医院”，在市区设立了惠民医院，在全市二级以上医院设立了惠民病房，卫生部对此给予高度评价。开展了济困助学“山花工程”[8]，筹捐1200多万元，资助贫困学生6679名。实施了农村饮水安全和“村村通”工程，130个村的饮水困难和饮水安全问题得到解决，5万贫困人口实现脱贫；新改建农村公路1384公里，基本实现村村通油（水泥）路。加强了文明生态村创建工作，新创文明生态村558个。推进了城建“三做”[9]便民工程，改扩建27条主次干道、4大路口、90条便民小路，改造33条街道路灯，天然气扩户1.17万户，集中供热面积新增163.6万平方米。为357户城镇最低收入家庭提供了住房保障。免费开放了竞秀公园和东风公园，城市绿化列全省第一档次。社区建设经验在全国推广。开展了“打黑除恶”、“打抓破”[10]等专项行动，打掉犯罪团伙556个、黑恶势力团伙52个，抓捕犯罪嫌疑人7001名，抓捕逃犯3668名，破获各类刑事案件22990起。民主法制和社会治安综合治理等工作进一步加强。事实表明，只要我们始终带着感情、责任和追求，真心实意地为群众办实事、办好事，就会赢得群众的拥护和支持，就会促进社会和谐稳定。

第四，改革创新狠抓工作落实。为确保对全市人民的庄严承诺落地生根，围绕落实《政府工作报告》，从制度建设入手，探索建立了一整套工作落实体系。一是建账。制定了《政府工作报告2006年落实方案》，将工作任务分解成461个具体事项，逐项明确了责任单位、责任人员和完成时限。把话落实到了事上，把事落实到了人上。二是明账。向市委、人大、政协专题报告，建议把《方案》作为对政府工作考核、监督的主要内容，将《方案》全文登报，接受社会监督。三是查账。通过现场督办、网络督办、明查暗访等多种形式，对落实情况进行动态跟踪问效。市政府班子成员下基层督导500多次，协调解决各类问题200多个。四是算账。制定了考核评价办法，结果与单位和干部的考核挂钩。建立并严格执行工作不落实责任记录备案制度，对5名县级和20名科级干部的不作为行为进行了责任追究，对11个单位的不作为记录备案、通报批评。这些措施有效地促进了工作落实，《方案》确定的461个具体任务完成453项，另外完成重大事项25项。实践证明，只要勇于改革创新，敢于把工作依法置于全社会监督之下，较真问责，抓住不落实的事，处理不作为的人，就一定能够把全市上下抓落实的积极性调动起来，把工作落实下去。

通过一年来的努力工作，全市发展能力进一步提高，社会稳定和谐的局面更加巩固，经济发展呈现稳中趋实的良好态势。全市生产总值完成1185.4亿元，增长10.4%；全社会固定资产投资578.6亿元，增长17.9%；全部财政收入98.4亿元，增长15.3%；地方一般预算收入42.6亿元，增长16.4%；社会消费品零售总额441.1亿元，增长14.5%；城镇居民人均可支配收入9632元，农民人均纯收入3686元，分别增长11%和6.2%。

各位代表，来之不易的成绩，是省委、省政府和中共保定市委正确领导的结果，是市人大、市政协监督支持的结果，是全市人民团结奋斗的结果。我们不会忘记，在拆违最艰难的时刻，人大代表和政协委员现场视察给了我们极大鼓舞。拆违中很多群众舍小家顾大家、主动拆除自家门脸；治理“三轮车”时车主师傅们顾全大局、主动配合；处置大沙河污染突发事件时广大干部群众连续奋战七昼夜；竞秀公园开放时老大爷、老大妈和中小学生主动担当环保志愿者；治理狗患时，街道社区的同志们走街进户、尽心尽责；1300多名大中专学生积极参加“山花工程”助学行动；新闻单位的同志们不辞劳苦，深入宣传报道，营造了良好的氛围，等等。这些事迹和情景至今历历在目，令人感动。每个难题的破解，每项工作的推进，都得到了全市人民的理解和支持，凝聚着广大干部群众的心血和汗水。在此，我代表市政府向全市人民，向驻保解放军、武警官兵和政法干警，向各民主党派、工商联、无党派人士和各人民团体，向所有关心和支持保定发展的海内外朋友，致以崇高的敬意和诚挚的感谢！

各位代表，特别需要报告的是，《政府工作报告2006年落实方案》中，争创双拥模范城“六连冠”等4项因上级推迟评比核定时间取消考核，地区生产总值、固定资产投资和启动建设文体、会展中心4项没有完成。去年国家发改委来我市检查新开工项目情况，发现有的县把续建报成新开，把小的报成大的。发现问题后，我们坚持正确的政绩观，坚决反对数字攀比和数字政绩，下决心解决数字不实问题，是多少就多少，要给全市人民一个实实在在的交代。这样做，也是要树立这样的导向，就是不能在数字上作文章，实事求是才是对人民利益的真正负责。在规划、启动文体中心和会展中心建设过程中，我们感到，当前保定最紧迫的是发展产业、发展生产力，要树立“舍得”理念，舍一时而求长远，舍一事而求全局。我们下决心将文体、会展和行政、商务“四大中心”[11]规划用地调整用于“中国电谷”建设。文体、会展中心将积极谋划，择地逐步建设。这样做，也是表明我们的决心，就是要把紧缺的资源要素优先用于发展产业，积蓄实力，真正为保定的长远发展负责。

一年来的工作实践，使我们对保定发展中的问题和矛盾有了更清醒的认识。从发展现状看，目前我市还处在由传统农业向现代农业转变、工业化初期向中期迈进、城市化进程刚刚起步阶段，经济总量小，总体水平低，发展后劲弱，遗留问题多，稳定任务重，处在爬坡过坎的特殊时期，面临“补课”和赶超的双重压力。特别是近几年主要经济指标增速在全省位次后移，去年有些指标排在全省后三位，形势非常严峻。如果再不奋发崛起，将被远远落下。深入分析，制约我市发展除了区位、人口等客观因素以外，重要的还在于体制和思想障碍。从体制障碍上讲，还没有形成适应经济快速发展和现代城市管理的体制机制，特别是地市合并后，体制、机制调整没有完全理顺，不能很好地适应管理“大保定”的需要，有时仍然用管理“小保定”的方式指导工作。对区的管理时有越位，区级职能不完善，责权利不统一；对县的指导时有缺位，规划指导不够，政策引导不够，资源统筹配置不够，没有很好地解决管什么、怎么管的问题，影响了县工作能动性的发挥。从思想障碍上讲，受传统农耕文化和皇城官文化影响，文化生态环境不利于市场经济的发育。小富即安、重仕轻商思想还一定程度上影响着人们的思想和行为。一些人封闭保守，开放意识、开拓精神不够；一些人热衷于围着官场转，盯着位子争，尊商、亲商的意识差；一些人总是自我感觉良好，爱贬低别人，缺少宽容、成全他人的胸怀，等等。这与我们为之自豪的文化底蕴不相称，在某些方面成为开放文明的禁锢，成为经济发展的阻滞力、对外开放的排斥力，在某种程度上也成了影响干部集中精力抓发展、影响群众干事创业的一种消极文化环境。

从政府工作上看，许多工作应该做好还没有做好，应该做到还没有做到，还存在不少问题和不足。主要有：一是全市上下抓经济、抓发展的氛围还没有形成。面对各地你追我赶的竞争态势，一些领导干部缺乏危机感，抢抓机遇的意识不强，干事创业的劲头不足，遵循市场经济规律抓发展的办法还不多、本领还不强，改革推进慢、招商效果不明显、龙头型大项目偏少等问题没有得到很好地解决。二是关系群众切身利益的问题解决得还不够好。一部分群众生活还相当困难，看病难、看病贵、上学难、上学贵等问题比较突出；不少困难家庭住房问题还没有解决；大学生和城镇新增劳动力就业难的问题日益显现；安全生产事故和食品、药品安全问题还时有发生；城市马路市场、小街巷卫生死角、公交车覆盖面窄等问题还没有很好地解决。三是新农村建设的推进步伐还不够快。作为农业大市，解决“三农”问题的长效办法还不多，农村基础设施、公益事业等方面还有很多欠账，特别是还缺乏促进农民可持续增收的有效措施。四是发展环境还没有得到有效改善。服务企业和群众的意识还不强，有人把应尽的服务当成“恩赐”，把手中的权力当成谋私的筹码，“吃拿卡要”现象在一定程度上还存在，外地客商意见大，本地企业不满意，提起来就是一肚子气。行政服务中心的设立使办事效率提高了不少，但要盖的“戳”还没减少。治安状况虽有好转，但还没有得到根本改善，群众还缺乏安全感。五是工作作风还需进一步转变。少数政府公务人员责任心不强，工作不实，甚至弄虚作假；铺张浪费现象还普遍存在，一些领域的消极腐败问题还比较严重，损害群众利益，影响党和政府形象。对这些问题，必须认真加以解决。

二、2007年目标任务

当前，我市正处在改革发展的关键期，既面临前所未有的挑战，更面临千载难逢的机遇。置身经济全球化深入发展的时代浪潮，面临承接产业转移、技术扩散，打造竞争新优势的难得机遇；置身我国经济社会步入科学发展的历史进程，面临转变经济增长方式、实现更好更快发展的难得机遇；置身环渤海地区加速崛起的发展大势，面临充分发挥“两环”区位优势、加快自身发展的难得机遇；置身我省建设沿海经济社会发展强省的奋进征程，面临接轨沿海、贯通东西，发展外向型经济的难得机遇。

面对新形势、新任务，我市明确提出了建设京南近海强市名城的奋斗目标。“京南”，就是凭借京南中心城市的优势，主动承接京津辐射，打造“南资北移”、“南企北扩”的桥头堡，力争在京津冀一体化发展中占有重要一席。“近海”，就是利用沿海一线腹地、“东出西联”中继站的特殊区位，融入沿海经济隆起带，积极参与国际经济大循环。“强市”，就是促进经济向大总量、高层次加快迈进，推动经济实力快速提升。“名城”，就是深入挖掘历史文化名城的深厚底蕴，重振古城辉煌。这一发展目标，符合我市市情和全市人民心愿，我们必须为之努力奋斗。

2007年政府工作的指导思想是：以邓小平理论和“三个代表”重要思想为指导，以科学发展观为统领，高扬“树正气、讲团结、求发展”主旋律，认真贯彻中央、省和中共保定市委的决策部署，紧紧围绕科学发展、提速发展、和谐发展主题，更加注重调整经济结构、转变增长方式，培育产业支撑和文化支撑，更加注重项目建设和发展县域经济，深化改革、扩大开放，更加注重资源节约、环境保护，统筹城乡和区域协调发展，更加注重发展社会事业、解决民生问题，坚定不移地朝着建设京南近海强市名城的宏伟目标迈进。

经济社会发展主要预期目标是：生产总值增长12%以上；单位生产总值能耗下降4.4%；主要污染物化学需氧量和二氧化硫排放量分别减少3.8%和2%；全社会固定资产投资增长20%以上；社会消费品零售总额增长12%；全部财政收入可比增长12%以上；地方一般预算收入可比增长12%；城镇居民人均可支配收入增长8%；农民人均纯收入增长6%；城镇登记失业率控制在4.5%以内；人口自然增长率控制在6.8‰以内。

今年工作的基本要求是夯实基础，狠抓落实，提速发展。夯实基础，就是进一步在科学谋划、改革创新、解决难题上下功夫，继续扫除发展障碍。狠抓落实，就是抓好“十一五”规划和去年各项具体工作思路的落实，努力把工作往深里做、往实里做。提速发展，就是以工作提速推动发展提速，以资源集中配置保障发展提速，能快则快，好中求快，又好又快。

做好今年工作，最根本的是用科学的思想方法、科学的执政方式、科学的发展模式推动发展，最关键的是抓住主要矛盾、突破发展瓶颈，最重要的是抢抓新机遇、打造新优势、狠抓工作落实。我们要下决心与一切影响改革、开放和发展的僵滞思想、落后文化、陈规陋习做斗争，努力破除阻碍发展的思想束缚；下决心解决发展的体制障碍，加快推进行政管理体制改革，建立科学的市县管理体制和现代城市管理框架；下决心把土地、资金、人才等资源要素优先配置到促进生产力发展上来，加快推进战略支撑产业发展，积蓄经济实力和发展后劲；下决心促进产业布局由分散转向聚合、产业发展由粗放转向集约，加快经济结构调整和增长方式转变；下决心解决群众最关心、最直接、最现实的利益问题，从群众身边的事情入手，真心实意为群众排忧解难。

三、推动经济提速发展

发展高于一切，实力决定一切。必须把迅速提升经济实力作为我市当前工作的重中之重、当务之急来抓。

第一，举全市之力推进工业强市。“保定制造”是我市优势所在、希望所在，必须全市聚焦、全面叫响。扎实开展“项目建设突破年”活动，以项目支撑做强产业支撑，构建特色鲜明的“3+4”主导产业[12]格局。

加快建设“中国电谷”。生态文明时代必然带来新能源产业的兴起，必将引发划时代的产业革命。建设“中国电谷”，对于加快我市工业化进程具有战略意义，是推动我市产业升级、实现跨越发展的出路所在。必须抓住近三、五年的关键时期，举全市之力，加快推进。要强化政策支撑，促进项目、技术、人才集聚。强化资本支撑，积极引进国际战略投资，加强与国家开发银行合作，建立“中国电谷新能源产业基金”。强化智力支撑，推进与华电、中科院合作迈出新的步伐。强化项目支撑，突出抓好投资30亿元的天威英利三期、投资4亿元的风帆太阳能电池、投资6亿元的惠腾兆瓦级叶片、投资2亿元的国电集团风电整机和投资8亿元的钢铁研究总院非晶带材等一批重点项目。全力打造光伏、风电、输变电设备、新型储能、高效节能、电力电子器件、电力自动化及电力软件等七大产业园区。力争用10年左右时间，在城区北部建成占地20平方公里、年销售收入超1000亿元的新能源和能源设备制造基地，建成国内外著名的“电字号”产业、技术、人才和信息聚集区，使保定成为中国新能源产业发展的领军城市。

加快建设“华北轻型汽车城”。我国汽车工业前景广阔。我们要坚定发展民族汽车工业的雄心壮志，聚合民营企业，打响民族品牌，实施国际化战略，坚持差别竞争、错位发展，建设走向世界的中国民族汽车工业生产基地。今年，要加快城区南部保定零部件生产基地建设，力争年内入园企业达到5家。长城三期竣工投产，实现长城轿车全球同步上市；中兴汽车出口基地建设达到美国标准，为明年出口北美做好准备；河北长安开发两个新车型，生产线竣工投产。全市整车生产能力达到70万辆，产量达到33万辆，出口保持全国领先。到2010年，全市整车生产能力达到100万辆，零部件企业发展到500家，汽车产业销售收入超过500亿元，建成全国SUV、皮卡、微卡、轿车生产和出口大市，成为华北著名的汽车及零部件生产基地。

加快建设“华北纺织服装产业基地”。发挥产业优势，着眼构建东部“一带、四城、十园”[13]纺织产业格局，突出抓整合、抓升级、抓治污，创优品牌，做强龙头。积极促进宏润纺织和北京雪莲的强强联合，加快大河服装等园区建设，抓好高阳精梳纱及高档面料、容城澳森制衣等产业升级项目。加大治污力度，加快高阳、白沟等污水处理厂建设。抓住北京建设“时装之都”的机遇，强力推动与北京纺织产业全面合作，使保定成为北京“时装之都”的加工生产基地。力争到“十一五”末，年销售收入超50亿元的产业集群达到5个以上，全行业年销售收入超过600亿元。

加快发展文化产业。全面落实“一纲一力四目”，加快文化大市建设，彰显保定鲜明的文化特色，打造文化保定。让文化的力量影响、渗透到社会各个层面，促进文化向工业、城建、餐饮、商贸等各个领域渗透延伸，以文化力提升经济力，真正使“保定文化”这只轮子的作用发挥出来。坚持以产业演绎文化、以文化提升产业，推进与河北大学的战略合作，启动建设“河北动漫产业基地”，推进与韩国合作的影像IT文化产业基地建设。培育产业龙头，扶持壮大一批“专、精、特、新”文化企业群，开发一批高档次、高质量特色文化产品。搞好旅游宣传促销，深化“迎奥运游保定”和“旅游细微服务”系列活动。精心打造白洋淀、野三坡等9大精品景区[14]，做优“跟着老电影去旅行”、历史文化名城游、百里太行风光游等精品线路，抓好涿州古街、地道战纪念馆、定州宝塔文化观光园等投资21亿元的21个项目建设，全力打造“红色旅游、文化古城、山水保定”旅游名片。

推动其他产业加快发展。壮大骨干企业，延伸产业链条，做强食品加工、电子信息等成长性产业以及建筑建材、轻化工等优势产业。把服务业作为提升综合竞争力的重要产业来培育，加快现代物流体系建设，发展金融保险、信息咨询和中介服务等现代服务业，推行电子政务，打造餐饮名片，推进特色市场上档升级，努力培育新的消费热点。

第二，统筹推进区域协调发展。坚持“做大中心、发展南北、保护两翼”，进一步落实城乡规划管理全覆盖，搭建合作平台，探索建立区域经济行政协调、利益补偿、评价考核机制，做强基础设施支撑，走经营城市道路，推动重点区域率先崛起。

努力构筑基础平台。全力推进总投资1006亿元的“电、水、路”项目建设。推进定洲电厂二期建设，加快南郊、涞源电厂前期工作，抓好电网建设。保障南水北调保定段顺利施工。抓好保沧、保阜、廊涿、张石等6条高速公路和京郑高速铁路建设，构建“三纵三横一环”[15]高速公路网，打造100分钟临港交通圈，打开“东出西联”通道，为建设现代陆港城市，接轨京津、接轨沿海创造基础条件。

做大做强中心城市。把产业发展放在城市发展的首位，以做大产业来做大城市，以产业兴旺带动城市繁荣。统筹“一城三星”规划建设，促进资源共享和产业配套，增强中心城市辐射带动力。建设中国北方独具文化特色的园林式、生态型、现代化宜居城市，提升中心城市形象。重点在“水”、“古”、“亮”上下功夫，实施水系建设、古城区保护开发和“太阳能之城”[16]建设三大工程。编制市区水系建设规划，改造排水管网，实施污水截流、河道清淤，努力实现市区河道还清。恢复西大街历史风貌，抓好光园、淮军公所等文物景点修缮，规划治理裕华路街廓环境，努力使老城区再现“历史之韵”。全面推进太阳能在照明、供热等方面的综合应用，突出抓好城市建筑轮廓线亮化和路灯改造，把保定的夜晚打扮得更加靓丽。

着力打造保南、保北两个区域次中心。在南部，重点整合定州、安国、望都、博野等区域经济板块，建设微型车生产基地和中药材、农产品加工基地。在北部，重点整合涿州、高碑店、定兴、容城、雄县等区域经济板块，培育接轨京津和临港的加工配套业、现代物流业，联手打造融入京津的“金三角”。

加快建设西部山区生态产业带。合理划分重点开发区、限制开发区、禁止开发区，大力推进生态保护和综合开发。培育生态旅游、生态农业和绿色食品加工以及矿业、建材等支柱产业，建设绿色屏障、发展绿色产业，积极探索出一条消除区域贫困和改善生态环境“双赢”的有效途径。

提升县域经济聚合力。积极开展“县域经济突破年”活动，坚持“一立一特一主三化”[17]，以城兴业、以业兴城，加快城镇化进程。把园区建设和县城建设作为县域经济发展的龙头来抓，引导产业向园区集中、园区向城镇集中，抓成一批立县富民大项目，培育壮大特色产业集群。加强规划管理和公用设施建设，增强县城承载吸纳能力。大力发展民营经济，实施“催生、助长、扶强”战略，抓好中小企业“成长和素质提升工程”，加强企业家队伍建设，支持百家成长型企业上规模、上水平。完善信用担保、创业服务、信用评价、人才培训四大体系，为企业发展、全民创业营造良好环境。

第三，扎实推进新农村建设。不遗余力支持农村，脚踏实地发展农业，满腔热情帮助农民。按照“一个中心”、“三大保障”、“四个一批”的思路，发展新经济、建设新环境、培育新农民、落实新政策，推动新农村建设健康发展。

坚持以发展农村经济为中心，努力增加农民收入。着眼于“高产、优质、高效、生态、安全”，深化农业结构调整，大力发展现代农业。突出抓好“草牛奶”、蔬菜提质增效和林果生态工程，加快形成一批现代农业生产基地。抓住“良种、技术、设施、推广”四个关键环节，抓好“一县一业一园” [18]科技示范。健全农业标准、检验检测和产地认证三大体系，加强重大动物疫病防治，不断提高农产品质量安全水平。坚持把食品加工作为推进农业产业化的关键来抓，加快顺平汇源果品加工扩建、高碑店康师傅方便面一期和望都蒙牛等食品加工项目建设，扶持天香、绿健等重点企业做大做强，争取把定州伊利发展成亚洲最大的液态奶生产基地。每个县（市、区）每年都要力争上1-2个食品加工项目。发展劳务经济，实施劳动力转移培训阳光工程，完善劳务输出网络，推进劳务输出组织化、规模化发展，引导农民“走出来”，年内向非农产业转移农村剩余劳动力20万人。

建立健全农村“三大保障”，进一步改善农民生产生活条件。健全新农村建设体制保障，完善农民生活基本保障，强化农村基础设施和社会事业保障。推进以乡镇机构、农村义务教育和县乡财政体制为主要内容的农村综合改革，协调推进集体林权制度、农业科技服务体系、种子管理体制等各项改革。巩固完善各种支农惠农政策，落实好四项补贴政策和粮食最低收购价政策。财政支农资金增量、用于农村建设的比重、直接用于改善农村生产生活条件的资金都要高于上年。大力推进“七项工程”。实施村村通油路全覆盖工程，新建公路1260公里，使219个行政村通上油路。实施村村通广播电视工程，解决200个自然村听广播、看电视问题。实施电力惠民示范工程，建设1个电气化县[19]、8个电气化乡、76个电气化村；规范灌溉电价，实行井井通电、户户持卡，切实减轻农民负担。实施饮水安全工程，解决氟超标、苦咸水和山区严重缺水问题，使123个行政村的8.6万群众喝上放心水。实施生态家园富民工程，建设农用沼气池4.8万个。实施“万村千乡”市场工程，建设改造标准化农家店800家。实施扶贫开发到村到户“细胞”工程，加大整村推进和社会帮扶力度，启动14个先行村和7000个示范户，解决3万贫困人口和2万返贫人口脱贫问题，让农民群众的日子一天天好起来。

协调推进“四个一批”，因地制宜搞好新农村建设。坚持产业强村、文化兴村、生态建村、民主治村，先行搞好规划，积极探索新农村建设方式。一是转变一批。位于城郊的村庄，与城镇一体规划、建设，加快农村向城市、村组向社区、农民向市民转变。二是提升一批。具有较强产业基础的村庄，联手周边村庄，集中规划、连片建设，向小城镇发展。三是改善一批。具备一定人口规模的村庄，改善人居环境，逐步发展为中心村。四是迁建一批。对少数地处偏远，人口较少，不宜生存和发展的村庄，积极引导其向城镇和有条件的村庄集中。把文明生态村创建活动作为重要载体，新建450个，巩固提高1532个，推动文明生态村向连线成片方向发展。

第四，加快推进改革开放。坚持把改革开放作为发展的根本动力，努力在一些重要领域和关键环节取得突破。

打好国企改革攻坚战。把今年作为“国企改革攻坚年”，坚持以产权制度改革为核心，加快优势企业战略重组和亏困企业全面退出步伐。抓好列入计划的45户企业的破产重组。力争用两到三年时间，完成列入国家规划的市属22户国有企业政策性破产，完成市、区属164户企业的改革改制。强化国有资产监管，完善业绩考核体系，加强对企业财务风险的防范管理。对国有资源、资产、资本、资金统筹运作，探索资源转化为资产、资产转化为资本、资本转化为资金投入的有效途径。

推进行政管理体制改革。力争在城市管理体制和市县管理体制改革上取得突破。坚持政事分开、政企分开，理顺政府部门管理职责，理顺行政事业单位资产管理体制。坚持重心下移、权责一致，界定市、区两级城市管理职责。借鉴省“扩权强县”办法，争取省支持，积极探索对县（市、区）“放权、让利、松绑”改革，努力做到该管的一定管好，该放的坚决下放，可备案的就不再审批。

深化财税金融等各项改革。探索建立区域财政补偿机制和异地投资税收分享办法，促进项目建设打破行政壁垒向重点产业带集中。推进财政综合预算管理改革，完善政府债务管理机制。建立政府投资项目和专项资金管理制度，严格立项、可研、招标、监理、公示和审计等各项程序。加强与市外、境外金融机构合作，加快城市商业银行组建步伐，推进农村信用社改制。探索推广担保平台、风险评估平台和信用评价三位一体运作方式，全市中小企业信用担保资金力争达到6.5亿元、形成35亿元以上的担保能力。推进事业单位、县级供销社和文化体制等各项改革。

努力提高开放水平。适应入世过渡期结束和环渤海地区加速崛起的新形势，强化大开放、大沿海意识。加强对外宣传，努力提高保定知名度。创新思路，改进方式，探索建立专业招商机制，落实招商引资激励政策。加强开发区、工业集中区建设，使其成为招商引资的重要平台。着眼产业优化升级，融入沿海，借力京津，积极推进与国内外大公司、大集团的合作。加大签约外资项目盯办力度，提高外资到位率。抓好天威英利海外上市，做好中兴汽车、巨力吊索具等企业上市筹备工作。加快转变外贸增长方式，抓好优势产品出口基地建设，优化出口产品结构。支持有实力的企业“走出去”。

加强与驻保大企业和高校战略合作。驻保大企业和高校涉及人口占市区人口的近一半，是我市重要的开放平台、项目平台、人才平台、信息平台和消费平台，要对驻保大企业和高校高看一眼、厚爱一层。建立政企、政校协调机制，认真帮助解决实际问题，创优发展环境。加强与大企业和高校的战略合作，使之真正成为我市对外开放、加快发展的主力军

第五，大力推进经济增长方式转变。实施可持续发展战略，推进资源节约型、环境友好型社会建设，努力走出一条具有保定特色的新型工业化道路。

推动自主创新。努力加大科技投入，启动建设汽车、太阳能、风电、纺织服装等一批工程技术研究中心。加强产学研相结合，推进关键技术攻关，以保定创造支撑保定制造。支持高新技术产业、现代农业和现代服务业等重点领域开展科技创新，造就一批知名企业和知名品牌。深入实施人才强市战略，加强人才队伍和创新团队建设，努力创造人尽其才、人才辈出的良好环境。

狠抓节能降耗。严格落实上级政府与下级政府、政府与企业双向目标责任制，推进“双百企业节能工程”，力争如期达标。严格执行节能节水标准，推广节能环保技术，突出抓好建材、化工、电力、纺织等重点行业和重点企业的节能工作。广泛开展节约型机关、企业、社区和社会创建活动，增强全民节约意识。

治理环境污染。严格落实环保责任制，狠抓主要污染物排放削减。严格实行强制淘汰、限期治理和排污许可证制度，加大对水体、大气、土壤污染和噪声、汽车尾气等污染的治理力度。严把项目环评关，严禁低水平重复建设和污染环境的项目上马，绝不以牺牲生存环境来换取一时的经济发展。编制生态发展中长期规划。启动白洋淀亚行贷款综合治理和国家水体污染控制专项治理项目，探索建立区域补水机制，一定把“华北明珠”保护好。推进国家环保模范城市和生态园林城市创建工作。

节约集约用地。切实加强耕地特别是基本农田保护，严禁违法违规用地、滥占耕地。深入开展土地整理，抓好“空心村”、“砖瓦窑”和工矿废弃地整治，促进工业用地向开发区和工业区集中。深化土地供给制度改革，统筹安排建设用地，优先保障重点产业、大额外资以及省市重点建设项目用地。

发展循环经济。抓紧制定循环经济试点技术指标体系和验收标准，在部分园区和企业开展试点。研究制定建材、化工行业发展循环经济的实施办法，制定和完善再生物资回收的政策措施，推进工业废弃物和废旧资源循环利用，推行清洁生产。

四、努力构建和谐保定

社会和谐是经济繁荣、事业振兴、人民幸福的重要保证。坚持把民生问题作为根本着力点，从群众亟待解决的问题入手，在全市实施18项民心工程，办好89件实事，使发展成果更多更好的惠及全市人民，让广大群众的幸福感随着经济发展而增强。

第一，千方百计为群众排忧解难。努力为每一名困难群众提供基本生活保障。城镇低保月补差额由人均68元提高到72元，农村低保月补差额由人均13元提高到18元。养老、失业、医疗、工伤等各项社会保险扩面新增9.7万人。成立助困金融服务中心，为弱势群体提供金融服务。建立市流浪儿童保护中心，不让一个孩子流落街头。关爱残疾人生活，抓好公共场所无障碍通行设施建设。做好老龄人口工作，加快老年公寓建设。努力不让一户困难家庭出现零就业。实施城镇零就业家庭就业再就业援助工程，确保“零就业家庭”长期“动态为零”。落实积极就业政策，努力开发就业岗位。加快市劳动就业服务中心建设。做好大中专毕业生、被征地农民、复员转退军人就业指导服务。全年城镇新增就业4.7万人，帮助2.7万名下岗失业人员再就业。努力不让一名百姓因贫困而看不起病。推进新型农村合作医疗制度[20]全覆盖，由4个试点县扩大到全部县（市）。实施卫生惠民和农民健康保健工程，办好惠民医院和惠民门诊，创建甲级社区医疗卫生服务机构30个，新增农民健康保健试点村110个，改扩建乡镇卫生院75所，81所“流动医院”义务巡诊2000个村。加强传染病、职业病预防控制，提高突发公共卫生事件应急处置能力。严厉打击收受药品回扣、“红包”和开单提成等不正之风，严厉查处医疗乱收费。努力不让一个孩子因家庭贫困而上不起学。全部免除农村义务教育阶段学生学杂费，对城乡贫困家庭学生免费提供教科书，对贫困寄宿生补助生活费。维修改造农村中小学校舍9.2万平方米。继续实施济困助学“山花工程”，救助贫困学生3000名。统筹教育资源，规范学校收费项目和标准，制止教育乱收费，切实减轻群众的教育负担。加快“职业教育发展全方位”进程，抓好高职高专示范学校建设，筹建职业教育集团，创建2-3所省级以上职教名校，推进人口大市向人力资源强市转变。

第二，深入推进城建“三做”便民工程。市区改造4个交叉路口，新建、改建6条主次干道、10条社区支路，整修15条便民小路；改造易积水路段排水设施；办好“百姓公交”，新增公交线路3条、调整10条，向社区延伸，让群众出行更方便。竣工经济适用房11.2万平方米，为623户城镇最低收入家庭提供住房保障；改造老住宅小区供水管网，“一户一表”水表改造5000户、电表改造3万户；新增集中供热面积150万平方米；新改建3个便民市场、12座垃圾台站、9个标准公厕，引导商场、酒店等公共服务场所明确卫生间标识并对外开放；加强小区物业管理服务，让群众生活更加舒适便利。开展市容环境“洁、美、绿、亮、序”综合整治百日行动。严禁私搭乱建，打好拆违第二战役。治理乱涂乱画，规范整治户外广告。坚决取缔马路市场，治理背街小巷乱堆垃圾，让群众身边的卫生环境更清洁。免费开放植物园，建设6块游园绿地，为群众提供休闲健身的好去处。

第三，大力弘扬和谐文化。倡导和谐理念，营造尊重差异、包容多样、相互欣赏、相互关爱的人际关系和社会氛围。大力弘扬以“八荣八耻”为主要内容的社会主义荣辱观，抓好“五五”普法，扎实开展社会公德、职业道德、家庭美德和民主法制教育。办好古城戏剧节、彩色周末等活动。深入开展文化、科技、卫生“三下乡”，加强社区和乡镇综合文化站（室）建设，丰富城乡居民业余文化生活。广泛开展全民健身活动，培养更多的优秀竞技体育人才。办好市第六届运动会，承办好省第六届农民运动会，在省十二届运动会上争创佳绩。倡导婚育新风，严格实行人口目标管理责任制，稳定低生育水平，大力提高人口素质。落实计划生育奖励政策，依法征收社会抚养费。深入开展“关爱女孩行动”，综合治理出生人口性别比偏高问题。认真落实民族宗教政策，增强民族团结。加强国防和民兵预备役工作，争创全国双拥模范城“六连冠”。积极开展和谐社区、和谐家庭和群众性精神文明创建活动，争创全国和谐社区建设示范市和全省社区建设先进市。

第四，维护社会安全稳定。落实安全生产网络责任制，抓好“安全生产双百日承诺”活动，开展煤矿、非煤矿山和烟花爆竹等10大专项整治行动，遏制重特大事故发生。加快危化企业搬迁，努力排除群众身边的“不定时炸弹”。整顿市场经济秩序，加强食品、药品安全监管，重点加强对名牌和地产老字号食品的监管保护，让群众买着放心、吃着安心。坚持依法信访，深入做好矛盾纠纷排查调处工作。加强社会治安综合治理，深入开展平安社区、平安乡村创建活动，重心下移、警力下沉、保障下倾，把社会治安措施落实到基层，让群众感到警察就在眼前、平安就在身边。继续开展“打黑除恶”和“打抓破”行动，严厉打击村霸乡霸、地痞流氓等黑恶势力及其保护伞，持续打击街头抢包、公交车偷钱、偷盗自行车、入室盗窃等犯罪行为，努力使本地的犯罪分子无藏身之处、外面的犯罪分子不敢来保定，努力创造安居乐业的社会环境，让老百姓有更多的安全感。

五、加强政府自身建设

推动“科学发展、提速发展、和谐发展”，对政府自身建设提出了新的更高的要求。我们一定坚持科学行政、民主行政、依法行政，加快职能转变，建设务实、高效、负责的政府。

第一，增强本领，努力提高领导经济社会发展的能力。加强学习型机关建设，提高全体公务员素质，实现素质与时代对接、本领与工作对接，努力成为推动科学发展的行家里手，以更加宽广的眼光和战略思维推进工作。自觉运用市场经济规则规范行为，更多地运用市场经济的办法推动发展，努力提高管理现代城市的本领。完善经济运行监测和预警体系，改革和完善政府绩效评价制度，从制度上保障和提高政府领导发展的能力。加快转变思想观念，努力破除墨守陈规的思想束缚，树立勇于创新的思想理念；破除小富即安的小农意识，树立敢于争先的精神风貌；破除封闭保守的落后心态，树立大开放、创新业、建设强市名城的共同理想和坚定信念，以思想的大解放推动事业的大发展。

第二，履职承责，全面推进效能革命，掀起责任风暴。当前，管理无效率的问题非常突出，办一件事群众跑来跑去、领导批来批去、部门转来转去，问题推磨打转，久拖难决，这种状况必须扭转。人为“创造”工作量，延长“审批链”，背后就是为权力寻租创造机会，实质就是腐败。强化“政务就是服务”、“公务员就是服务员”的理念，把为纳税人、为群众服务作为价值观念来强化，作为行为准则来遵循，主动围着百姓干、围着企业转，严厉查处“吃拿卡要”行为，为更好更快发展创造良好环境。进一步削减审批事项和环节，优化规范办事流程，大力推行一次答复、限期办结制，推进政务提速。抓好市、县两级行政服务中心建设，没进厅的必须进厅，进厅的决不允许“只挂号不看病”，相关单位必须对“窗口”放权到位，决不允许“体外循环”。把今年作为“各项工作落实年”，建立健全抓落实的制度，落实事项都要分解到部门，落实到人头，明确工作标准和完成时限。突出危机导向和监督评价，把明责、履责、问责和奖惩紧密结合起来，严格不落实责任记录备案制度，强化“买单制”，失责必究。我们不怕得罪人，不怕丢选票，不怕引火烧身，决不允许占着岗位不作为、乱作为，有一个就处理一个。

第三，阳光行政，推进权力公开、透明运行。阳光是腐败的克星。大力推行政务公开，让公众的眼睛监督我们。利用市长短信平台、专线电话、政府网站等多种媒介，畅通民情民意反映渠道。坚持科学民主决策，完善重大事项专家咨询、集体决策、社会公示听证及新闻发布制度，邀请人大代表、政协委员和基层群众旁听政府决策会议，让群众享有更多的知情权、参与权和监督权。政府及其各职能部门的工作职责、“权力清单”、政务运作流程，除涉密事项外，全部向社会公开，从机制上不留下暗箱操作的机会和推诿扯皮的借口，规规矩矩为群众把事情办好。

第四，改进政风，切实做到团结、廉洁、节俭。加强官德政风建设，做人简单，做事务实，大力倡行胡锦涛总书记提出的“八个方面的良好风气”。把团结作为一种能力来锻炼和提高，作为一种文化来继承和弘扬，作为一条纪律来遵守和执行，发扬团队精神，形成发展合力。严格执行党风廉政建设责任制，加强监察、审计，坚决查处腐败。对那些以权谋私、官商勾结、贪赃枉法、腐化堕落的案件，抓住不放，一查到底，决不容情。始终牢记“两个务必”，厉行节约，坚决防止行政成本不合理上升。当前，广大群众对奢侈浪费、公款消费现象深恶痛绝。“一瓶酒够一个孩子一年的学杂费，一桌饭够一个贫困家庭一年的吃喝”，挥霍人民的血汗钱要有罪恶感！我们要自觉用良心、纪律约束自己，坚决抵制这种歪风邪气。

第五，求真务实，坚决反对造假做秀。现在，一些干部特别是有些领导干部心浮气躁、华而不实，突出表现为造假做秀。必须严厉整治这股歪风。要把求真务实的要求落实到各工作环节，落实到每名公务员身上。力戒浮躁情绪，防止急功近利，以对人民、对事业、对未来负责的态度，坚决反对“数字政绩”，严厉追查造假之事，处理做秀之人，让“苦干不如汇报，实干不如虚报”失去市场，使各项工作经得起历史和人民的检验。

各位代表，政府的一切权力是人民给的，我们一定自觉接受人大及其常委会的法律监督和工作监督，一定自觉接受人民政协的民主监督，一定认真听取各民主党派、工商联、离退休老干部和社会各界人士的建议和意见，主动加强与工会、妇联、共青团等人民团体的联系，不断改进政府工作，履行好政府的职责，决不辜负全市人民的信任和期望。

各位代表，建设京南近海强市名城，是全市人民的共同愿望，各级政府责任重大、使命光荣。我们深深的知道，实现这一目标任重道远，需要我们和衷共济、坚定信心、脚踏实地。必须认清保定当前发展形势不容乐观，发展任务相当艰巨，既要抓紧抓好当前工作，又要做好长时期艰苦奋斗的思想准备；必须营造团结和谐的政治环境，凝聚各方面力量，形成强大的合力；必须树立信心，以不屈不挠的顽强意志，勇敢面对前进道路上的困难和问题，不畏艰难险阻，敢于攻坚克难；必须拿出行动，埋头实干，一点一滴抓落实，一步一个脚印把工作推向前进。任何困难都不能阻挡保定人民创造幸福生活的美好愿望，任何挑战都不能阻挡我们建设京南近海强市名城的前进步伐。让我们更加紧密地团结在以胡锦涛同志为总书记的党中央周围，在省委、省政府和中共保定市委的正确领导下，热爱保定，建设保定，奉献保定，推动保定发展翻开新的辉煌篇章！
